# Transcatether Aortic Valve Implantation to Treat Degenerated Surgical Bioprosthesis: Focus on the Specific Procedural Challenges

**DOI:** 10.3389/fcvm.2022.895477

**Published:** 2022-05-31

**Authors:** Cristina Aurigemma, Francesco Burzotta, Rocco Vergallo, Piero Farina, Enrico Romagnoli, Stefano Cangemi, Francesco Bianchini, Marialisa Nesta, Piergiorgio Bruno, Domenico D'Amario, Antonio Maria Leone, Carlo Trani

**Affiliations:** ^1^Institute of Cardiology Fondazione Policlinico Universitario A. Gemelli IRCCS, Rome, Italy; ^2^Università Cattolica del Sacro Cuore, Rome, Italy

**Keywords:** valve in valve, degenerated surgical bioprosthesis, post procedural gradient, coronary occlusion, cerebral embolization, stentless aortic bioprosthesis, sutureless aortic bioprosthesis

## Abstract

Actually transcatheter aortic valve implantation within failed surgically bioprosthetic valves (VIV-TAVI) is an established procedure in patients at high risk for repeat surgical aortic valve intervention. Although less invasive than surgical reintervention, VIV-TAVI procedure offers potential challenges, such as higher rates of prosthesis-patient mismatch and coronary obstruction. Thus, optimal procedural planning plays an important role to minimize the risk of procedure complications. In this review, we describe the key points of a VIV-TAVI procedure to optimize outcomes and reduce the risk of procedure complications.

## Introduction

**Figure 1:**
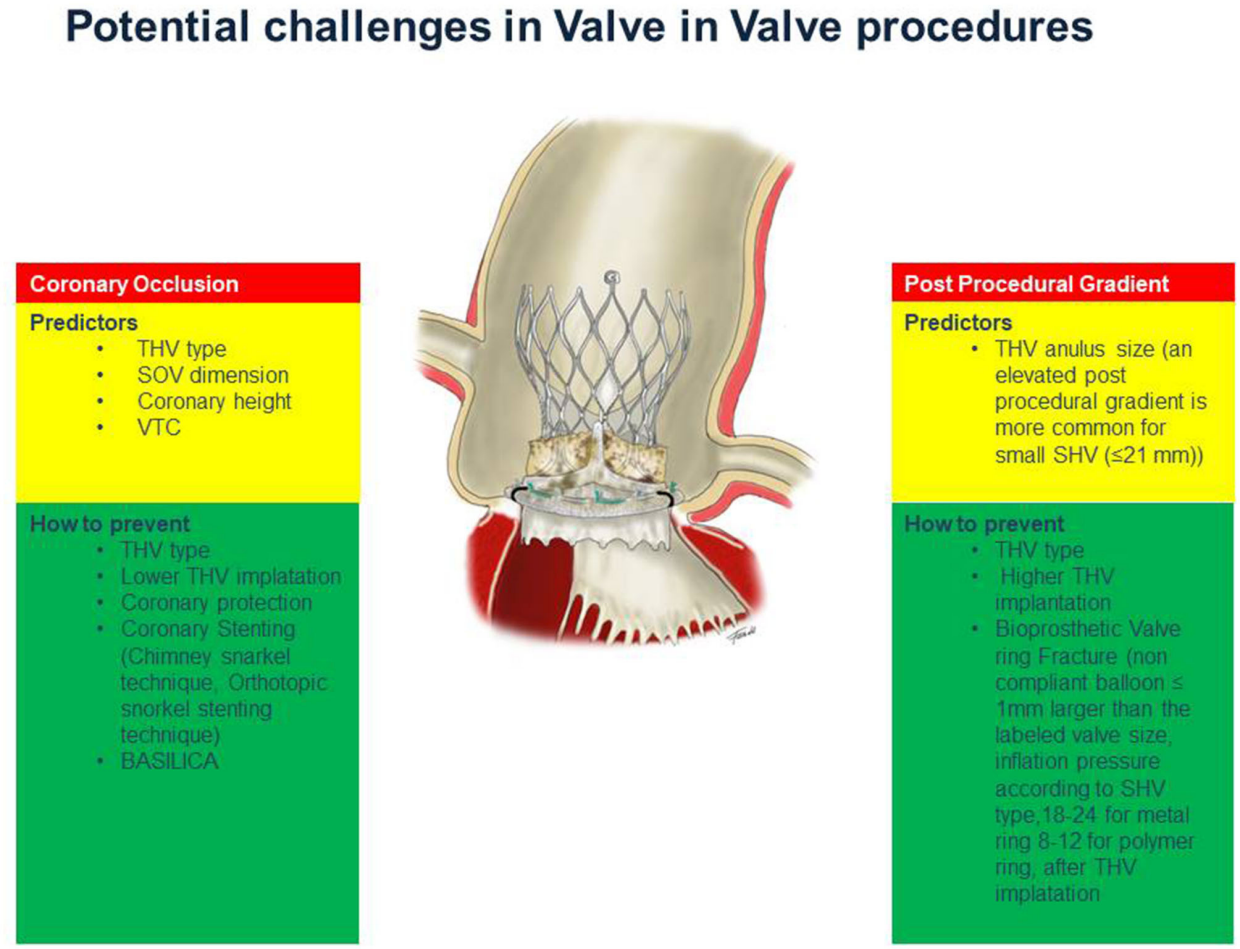
The key points of a VIV procedure to optimize outcomes and minimize the risk of operative complications.

Transcatheter aortic valve implantation (TAVI) is nowadays an alternative to surgical aortic valve replacements for the treatment of severe symptomatic native aortic valve stenosis (1–5). Moreover, several studies have also demonstrated that TAVI within failed surgically inserted bioprosthetic valves (valve-in-valve, VIV) is technically feasible (6–11). The first Valve in Valve (VIV) procedure was performed in 2007 in Germany (12), for severe aortic regurgitation in degenerated prosthesis, and actually transcatheter VIV-TAVI is considered an option for treating failed bioprosthesis in patients with increased surgical risk (13, 14). Recently a large USA retrospective study, enrolled 6.769 procedures, has demonstrated that VIV-TAVI was associated to lower in-hospital mortality but higher all-cause readmission at 30-day and at 6-month follow up compared to repeat surgical aortic valve replacement (SAVR) (15). However, no randomized trials have investigated the best treatment for failed surgically bioprosthetic valves comparing redo SAVR and VIV-TAVI. Therefore, clinical condition, surgical valve type and anatomy features should be considered to evaluate the better treatment between reoperative SAVR vs. VIV-TAVI to treat failed bioprosthesis (16). Although VIV-TAVI procedure is a natural evolution of the TAVI procedure, the potential challenges of the two procedures are different. In this review, we describe the key points of a VIV-TAVI procedure to optimize outcomes and reduce the risk of operative complications (Figure 1).

### The Different Surgical Heart Valves and the Concept of “True ID”

**Figure 2:**
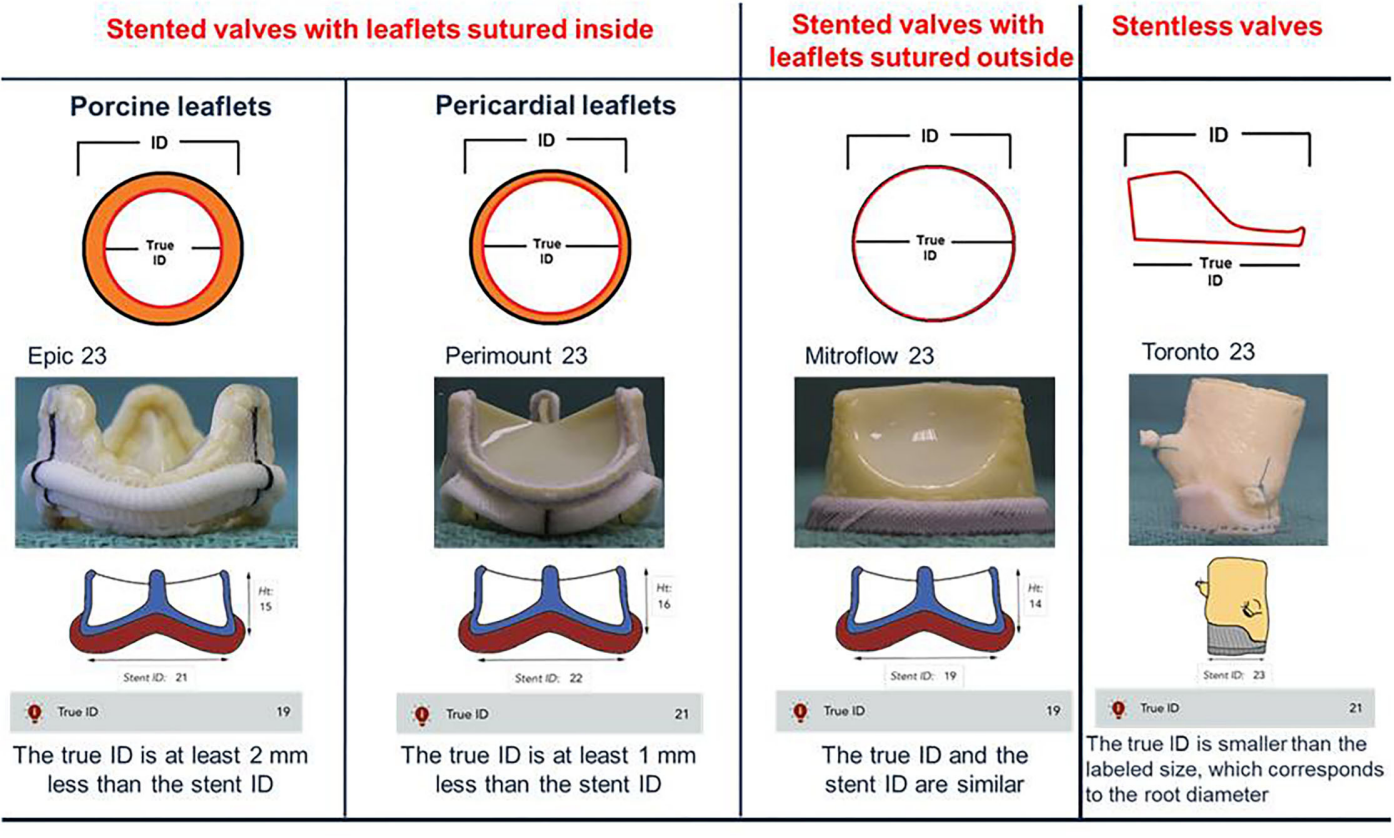
True internal diameter (ID) of stented and stentless surgical aortic valves. In stented valves with porcine leaflets sutured inside (Epic) true ID is at least 2 mm < the stent ID. In stented valves with pericardial leaflets sutured inside (Perimount) true ID is at least 1 mm < the stent ID. In pericardial valves with leaflets sutured outside (Mitroflow) true ID is the same as the stent ID. In the stentless valves (Toronto), which do not possess a rigid stent frame, the true ID is always smaller than the labeled size, which corresponds to the root diameter.

In TAVI procedure, the size of transcatheter heart valve (THV) is focused on the measurements performed at the level of the native aortic annulus (14). Instead during a VIV procedure, the internal diameter (ID) of the surgical heart valve (SHV) is used to select the appropriate THV size (8, 17, 18). In the setting of a correct sizing an important concept is the definition of the “true ID”. The true ID is the ID of the SHV inflow and the SHV design influences the true ID measurement (19). In stented valves the type and arrangement of the leaflets make the true ID smaller compared to ID of the stent frame. In stented THV with porcine valve leaflets sutured inside of the stent frame, the true ID is at least 2 mm less than the stent ID. Instead, in THV with pericardial leaflets sutured inside the difference between true ID and stent ID is less, about 1 mm. In pericardial SHV with leaflets sutured outside the true ID is similar to stent ID. In the stentless SHV, bioprosthesis without a rigid stent frame, the true ID is always smaller than the labeled size and corresponds to the root diameter (Figure 2). Furthermore, the stentless SHV can be surgically implanted in subcoronary placement or in full root replacement. The nature of the original surgical implantation is important because it may be subject to different challenges during a VIV procedure (see dedicated paragraph).

### Elevated Post-procedural Gradients

Although VIV-TAVI procedure restores valve function, a THV underexpansion, due to non-elastic surgical valve ring, is major limitation of VIV-TAVI implantation. In the Valve-in-Valve International Data (VIVID) Registry an elevated post-procedural gradients and severe prosthesis–patient mismatch (PPM) has been reported in 26.8% and it is more common with balloon expandable devices compared to self-expandable devices and in small surgical valves (≤ 21 mm) without the use of bioprosthetic valve ring fracture technique (20). In the PARTNER 2 (Placement of Aortic Transcatheter Valves 2) registry for VIV transcatheter aortic valve replacement (VIV-TAVR), unlike previously multicenter study results and VIVID registry data, there are no association between elevated echo mean gradient (≥ 20 mm Hg) or severe PPM and mortality at 3-year follow-up, suggesting that only severe PPM post-VIV may affected mortality (21). However the impact of suboptimal THV leaflet coaptation, leaflet-frame contact, and poor hemodynamics on device durability should be considered especially in patients with reasonable life expectancy. Therefore, a lower gradient after VIV-TAVI is an important target for this procedure. A better leaflet function and hemodynamics results may be achieved using THV device with supra-annular valve position. Indeed the function of THV leaflets positioned above the failed surgical valve ring is not hindered by the non-elastic portion of the original surgical valve. A vitro study have demonstrated that in a small failed surgical bioprosthesis (a 19-mm stentd aortic bioprosthesis) a supra-annular implantation of a THV is associated with a reduced postprocedural gradients and increased effective orifice area (22). In a study of 292 consecutive patients, a high implantation depth inside failed bioprosthetic has been demonstrated a strong independent predictor of lower postprocedural gradients in both self- and balloon-expandable transcatheter valves. According to this study an optimal implantation depths were 0 to 5 mm for CoreValve Evolut, and 0 to 2 mm (0–10% frame height) for Sapien XT (23) (Supplementary Figure 1). The bioprosthetic valve ring fracture (BVF) with high-pressure balloon inflation represents another technique to optimize hemodynamic results in patients with small failed bioprosthetic valves. The BVF facilitates THV expansion, maximizing the effective orifice area, and minimizes PPM. The minimum inflation pressures necessary for valve ring fracture are slightly different according to SHV type. In particular, in SHV with metal ribbon ring (i.e. Magna and Magna Ease) the fracture threshold (18–24 atm) is higher than SHV with a polymer ring (i.e. Biocor Epic, Mosaic, Mitroflow; 8–12 atm) (24). The high-pressure balloon inflation during BVF performed after THV implantation may cause structural damage to the self-expanding valve frame or leaflets, resulting in severe acute valvular regurgitation. A correct size of the balloon, a balloon smaller than the constrained segment of the self-expanding THV, and position, a balloon shoulder lower (i.e. more ventricular) than where the leaflets are anchored to the frame, can largely avoided this situation (25). The fracture of small surgical valves can be performed using both Atlas Gold and True Dilatation balloons, as demonstrated in bench testing and clinical experience (25). In bench testing and in the majority of clinical cases, balloons sized 1 mm larger than the labeled valve size were utilized. However in clinical setting smaller balloons were used successfully. Indeed balloons lager than the internal diameter of the SHV are able to fracture the valve, especially if a THV is already implanted (26). The timing of BVF, before or after THV represents an important question. A lager-sized prosthesis can be obtained and used with a BVF before THV implant, whereas, a further expansion of the THV itself can be performed with a BVF after THV implant. In the scenario of BVF before THV implantation, the resultant aortic insufficiency, such as the potential dislodgement and embolization of debris represent important concerns. On the other hand, if BVF follows THV implantation, an acute structural damage or accelerated degeneration of THV prosthesis itself should be considered as consequence of high-pressure balloon inflation. Recently an ex vivo bench test have demonstrated that BVF performed after THV implantation results in improved residual gradients (27), but the potential early and accelerated degeneration of THV device is not yet investigated. In conclusion, a BVF is considered to avoid post-procedural gradient after a VIV-TAVI, great attention should be paid to the choice of the balloon dimension and position. When the BVF is performed before THV implantation, the THV should be ready for timely implantation in case of exceptionally severe insufficiency.

### Coronary Occlusion

**Figure 3:**
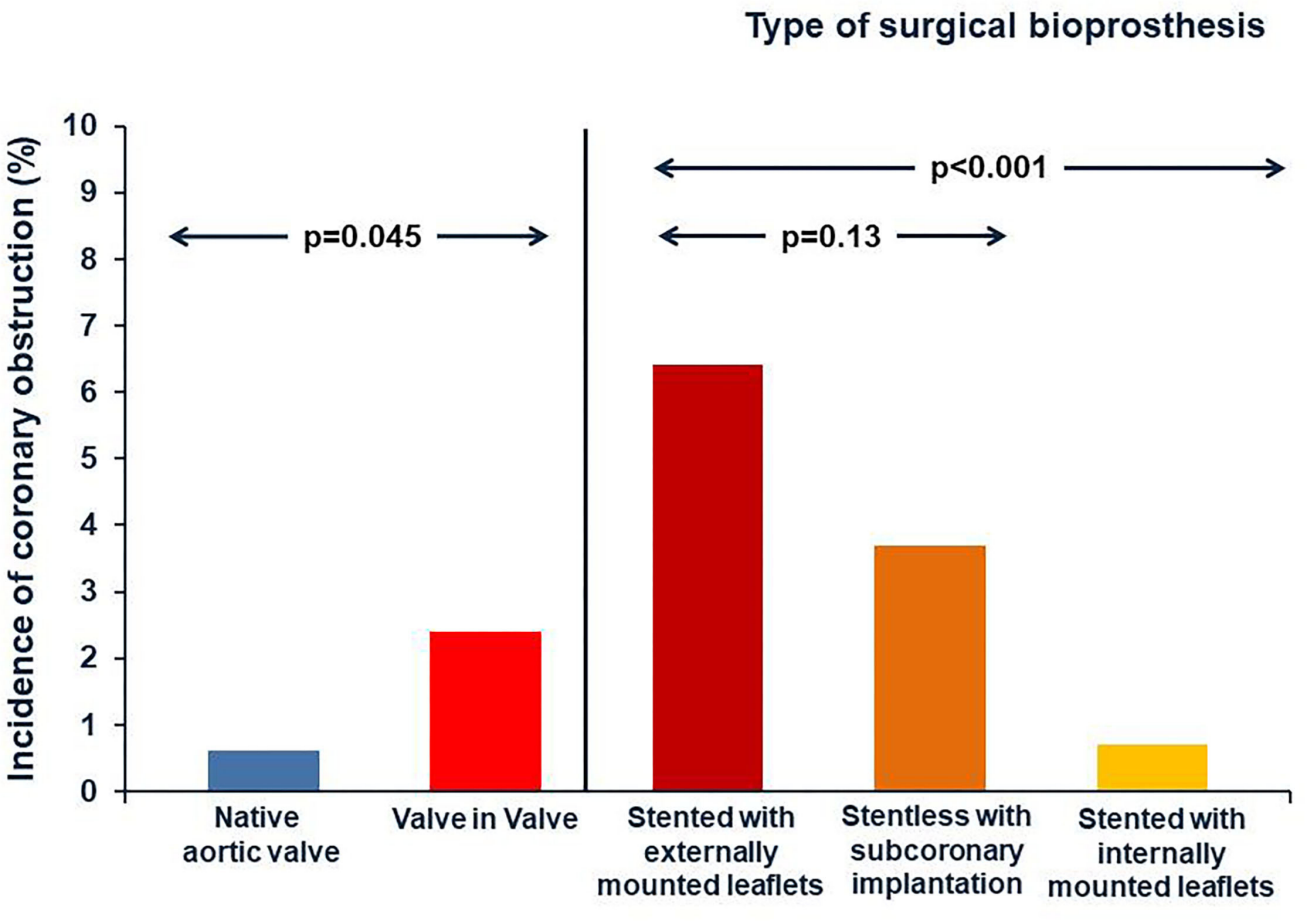
Incidence of coronary obstruction. The incidence of coronary obstruction is four folders greater in TAVI for degenerative bioprosthetic valves compared to TAVI for native aortic valves. The risk of coronary obstruction is also correlated to the type of SHV. Indeed it is highest during VIV TAVI procedures for surgical bioprothesis designs intended to maximize effective aortic orifice area (such as “stented” bioprostheses that have externally mounted leaflets, and “stentless” surgical bioprostheses).

During a TAVI procedure the incidence of coronary obstruction is <1%, it is a rare but life-threatening complication. The displacement of native valve leaflet toward the coronary ostia is the most common mechanism of coronary obstruction in TAVI procedure (28). This complication is more frequent during VIV-TAVI procedures. Indeed according to data of the VIVID Registry, the incidence of coronary obstruction is four folders greater in TAVI for failed bioprosthetic valves compared to TAVI for native aortic valves (29). The risk of coronary obstruction is also correlated to the type of SHV. Indeed VIV-TAVI in failed surgical bioprothesis designs intended to maximize effective aortic orifice area (such as “stented” bioprostheses with leaflets mounted externally, and “stentless” surgical bioprostheses) is associated to highest incidence of coronary obstruction (30) (Figure 3). Specific anatomic factors such as low coronary ostium height and small Valsalva sinus size are associated with coronary occlusion, as in TAVI for native aortic valve. In VIV-TAVI procedure another important predictor of coronary occlusion is the virtual transcatheter valve to coronary ostium distance (VTC). The VTC is the distance between the virtual ring designed into the diameter of the fully expanded THV and the coronary ostium (Supplementary Figure 2). Therefore, the VTC distance considers the sinus diameter and the coronary ostia height, but it is also influenced by the relative orientation of the bioprosthesis in the aortic root (31). A shorter VTC distance predicted the coronary occlusion complication, with an optimal cut-off level of 4 mm (32). The Valve-inValve International Data (VIVID) registry investigators have recently proposed a simplified classification that may guide operators on the risk of coronary obstruction during TAVI (33). In this classification three types of anatomy are identified:

-Type I anatomy, the failed valve leaflet extends fully below the coronary ostia plane and therefore the risk of coronary obstruction is close to none;

- Type II anatomy, the failed valve leaflet may extend above part of the coronary ostium, but not near the sinus tubular junction (STJ). If the sinus has large capacity to accommodate the deflected failed valve leaflet the risk of coronary obstruction would be low (Type IIA) on the other hand in presence of small sinuses (VTC distance is <4 mm) the risk of obstruction would be high (Type IIB);

- Type III anatomy, which the failed valve leaflet can extend either above the STJ plane, or below the STJ plane but very near it (<2 mm). If the virtual transcatheter heart valve to sinotubular junction (VTSTJ) distance is sufficient to allow diastolic flow to the coronaries the risk of coronary occlusion is low (Type IIIA), indeed in presence of the VTC <4 mm the risk of coronary occlusion is high (Type IIIB).

When the risk of coronary occlusion is high some procedural strategy may be considered. The deliberate implantation of a smaller diameter THV or underfilling and thus underexpansion of a balloon expandable THV reduces the lateral displacement of surgical valve posts and leaflets and consequently coronary obstruction. Similarly, a low depth THV implantation within the bioprosthesis induces less outward displacement of the surgical valve posts and leaflets compared to a high depth implantation, although postprocedural gradients may be higher in these cases. The type of THV is also relevant. In VIV-TAVI procedure with high risk of coronary occlusion the use of a recaptured self-expandable THV device is advantageous. Indeed the deployment of these THV devices could be followed by clinical and angiographic assessment of coronary flow status before the complete release and in case of coronary flow impairment, the THV device could be retrieved from the aortic root with relief of the coronary obstruction. Some THV devices have unique clipping mechanism that may prevent coronary obstruction by grasping surgical valve leaflets and attaching them firmly to the THV device. A recent bench test, in which THV was placed within stented SHV with leaflets mounted outside, have demonstrated that implantation of THV that interact directly with the surgical valve leaflets results in retraction of these leaflets decreasing the risk for coronary obstruction (32). In VIV-TAVI procedures with high risk of coronary occlusion the placement of a wire and a stent in the coronary artery is a more controlled preventive measure. Specific technical considerations may be performed in the choice of coronary intervention equipment used in these cases. The guiding catheter used to approach the coronary ostia from above should not interfere with THV device implantation (ie, Judkins left vs. Extra back up). A short tip guiding catheter can be preferred in order to be more easily pulled out of the coronary ostium during THV implantation. Furthermore, in many cases, the guide catheter could be used instead of the pigtail during THV device implantation (34). In cases of coronary obstruction the stent may be implanted according to the chimney snorkel stent technique. According to recent study the presence of adequate coronary flow after deployment might be not enough to decide against coronary stenting, a delayed coronary occlusion after VIV-TAVI procedures with high risk features have been reported. Therefore, the authors recommend a low threshold for stent deployment (35). In this setting we also consider that prophylactic chimney/snorkel stent technique is a potential predictable stepwise method of coronary protection (Table 1) but long-term durability of a stent release under the valve structure is of course pending (36). A coronary stenting through the prosthesis valve frame structure (coronary recannulation and wiring after valve release) according to the orthotopic snorkel stenting technique (Table 1) is recently proposed (37) (Supplementary Figure 3). An emergent preventive measure of coronary occlusion is the bioprosthetic or native aortic scallop intentional (BASILICA) (38). A prospective multicenter study enrolled 30 patients have demonstrated that BASILICA was feasible in native and bioprosthetic valves (39) and nowdays this technique is also proposed to prevent coronary obstruction in TAVI in TAVI procedure (40). However it cannot be ignored that this technique requires a learning curve and is associated with a not negligible stroke rates even in experienced centers (39).

**Table 1:** Steps, advantanges and diadvantages of the stenting techniques during coronary protection in VIV-TAVI procedures.

**Chimney Snorkel Stenting Technique**	**Orthotopic Snorkel Stenting Technique**
**Technique Description**	**Technique Description**
• Guiding catheter, wire(s) and stent are positioned in coronary artery prior to percutaneous balloon aortic valvuloplasty or THV deployment	• Guiding catheter, wire(s) and stent are positioned in coronary artery prior to percutaneous balloon aortic valvuloplasty or THV deployment
• The stent length selection: the stent comes above the height of the pre-existing bioprosthesis *in situ* (with enough stent in the proximal port of the coronary artery to allow for adequate anchoring) and above the most superior portion of the THV bioprosthesis leaflet or commissural attachment point	• The baseline stent length is selected according to chimney/snorkel stenting technique
• Eventual THV post-dilatation is performed prior to coronary artery stents deployment	• Eventual THV post-dilatation is performed prior to coronary stent decision
• Coronary stent is deployed with a substantial portion of the stent hanging into the aorta and ideally at least enough to come above the highest tract of the sealed portion of the THV	• After the prosthesis implantation and eventual post-dilatation, if coronary is not completely occluded, a second guiding catheter is advanced into the THV to reach the coronary ostium thought the prosthesis frames and the coronary artery is wired.
• Stent balloon pulled back away from the distal edge is inflated to higher pressures for flaring the proximal stent improving chance of re-access	• The stent is advanced and positioned from the coronary artery to the THV prosthesis with minimal protrusion inside the frame
• A kissing technique with simultaneous inflation of the THV balloon and the coronary stent balloon can be performed but is not mandatory	• Stent balloon pulled back away from the distal edge is inflated to higher pressures for flaring the proximal stent improving chance of re-access
**Technique advantages**	**Technique advantages**
• Quickly coronary flow restoration withdrawing and deploying the coronary stent in case of coronary occlusion	• A physiologic THV frame/coronary stent configuration with reduced external stent compression risk and facilitate coronary recannulation
**Technique disadvantages**	**Technique disadvantages**
• No physiological and very complex THV frame/stent configuration with possible coronary stent compression	Higher technical complexity and increased procedural time
Repeated coronary angiography or interventions may be more difficult	• The THV prosthesis orientation influences the procedure

### Cerebral Embolization

Cerebrovascular accidents (CVA) including stroke or transient ischaemic attack represent one of the most feared complications of TAVI procedures. Although the pathogenesis of the CVA following TAVI is likely multifactorial, embolization is likely to be the dominant mechanism. Aortic plaque, valve disruption during devices passages, thrombus formation during the procedure, and subacute thromboembolism originating directly from the native-THV or caused by chronic or onset atrial fibrillation represent the main source of emboli (39). New silent cerebral ischaemic embolic lesions, involved the two cerebral hemispheres and circulation territories, were found in up to 80% of patients who have undergone TAVI. However, only 3–6% of patients have showed new persistent clinical neurological impairment (41–43). Cerebral embolic protection devices (CEPDs) were introduced to reduce the risk of CVA and silent emboli, preventing that procedural debris reach the cerebral vasculature. The use of CEPDs is associated to a reduction in cerebral lesion volume without a substantial reduction in post-procedural or 30-day stroke and/or 30-day mortality (44). In the VIVID Registry the reported incidence of procedural major stroke was 1.7% (20). Therefore, the VIV-TAVI procedure is not associated to higher incidence of cerebral events compared to TAVI procedure in native aortic valve. However, the presence of much degenerated leaflets and the possible BVF pre-THV implantation might increase the risk of CVA and the use of CEPD might be consider in the planning of these procedures (Supplementary Figure 4).

### Particular Demanding Setting: VIV-TAVI in Stentless and Sutureless Degenerated Valve Prosthesis

The VIV-TAVI is an emerging, safe and reliable treatment option for degenerated surgical bioprostheses, VIV-TAVI procedures in stentless and sutureless bioprotheses have yet remained demanding procedures (Table 2). Whereas, a reduced risk of annular rupture, a lower rates of permanent pacemaker implantation, and a reduced paravalvular leak are reported in stented VIV-TAVI procedures when compared with native aortic valve TAVI procedures (34), the stentless VIV-TAVI treatments are associated to technical challenges and potential procedural complications. Indeed in a retrospective analysis of VIVID registry data procedural complications, such as device malposition, a second THV, coronary obstruction, and paravalvular leak, were more frequent during stentless VIV-TAVI procedures compared to stented VIV-TAVI procedures, but no difference in 30-day and in 1-year outcomes were found (45). The lack of radiographic and anatomic landmarks and the need to anchor on the non-compliant Dacron ring, which is sewn to the annulus below the nadir of the stentless valve leaflets, represent challenges for the correct position of the THV. Furthermore, leaflet prolapse is the more common cause of stentless surgical heart valves fail with consequent severe aortic regurgitation as predominant failure mechanism. Therefore, the absence of calcifications represents an additional problem for the THV anchoring. Coronary obstruction is more common in stentless VIV-procedure compared to native aortic valve or stented valve with leaflets mounted inside. The risk of coronary occlusion is higher in subcoronary implantation of stentless prosthesis. Indeed a subcoronary surgical implantation is associated with a shorter linear distance between the nadir of the stentless valve leaflets and the coronary arteries, as well as a shorter VTC distance, when compared to a full root implantation. In stentless with subcoronary implantation a THV position below the nadir of the stenless valve leaflets is associated with a reduction of the risk of coronary occlusion (46).

**Table 2:** Main challenges of stentless and sutureless VIV-TAVI procedures.

**Stentless VIV-TAVI procedures**	
Features	Procedural complications
• Lack of radiographic and anatomic landmarks • Anchor on the non-compliant Dacron ring • Absence of calcifications due to predominant leaflet prolapse and severe aortic regurgitation as failure mechanism • Subcoronary implantation of stentless prosthesis is associated to shorter linear distance between the nadir of the stentless valve leaflets and the coronary arteries, as well as a shorter VTC distance	• Higher risk of device malposition, a second THV, paravalvular leak • Higher risk of coronary occlusion
**Stentless VIV-TAVI procedures**	
Features	Procedural complications
• Elastic structure of the sutureless valve • Absence of sutures • An upper ring is located at the level of the decalcified annulus, while the lower segment of the valve protrudes in the left ventricular outflow tract for about 5 mm	• Valvular instability and dislocation when a THV is implanted inside • An incomplete expansion of the THV prosthesis implanted too low

Whereas, the successful VIV-TAVI treatments have been reported in patients with degenerated sutured stented or stentless valve prosthesis, few data are available to demonstrate the feasibility of VIV-TAVI procedures in degenerated sutureless aortic valve prosthesis (47–49). Among VIV-TAVI procedures the sutureless VIV-TAVI offers additional challenges. The elastic structure of the sutureless valve stent and the absence of sutures can theoretically increase the risk of valvular instability and dislocation when a THV is implanted inside. The sutureless valve is characterized by two rings (one lower and one upper), three commissural elements supporting the valve and three pairs of sinusoidal elements fixing to Valsalva sinus. When the sutureless valve is correctly positioned, the upper ring is located at the level of the decalcified annulus, while the lower segment of the valve protrudes in the left ventricular outflow tract for about 5 mm. A THV positioned too low can result in an incomplete expansion of the THV prosthesis with leaflets malfunction due to the constriction by the sutureless nitinol ring. Consequently, the THV should be positioned at the level of the lower edge of the sutureless valve or 2–3 mm higher. In sutureless VIV-TAVI procedure the cause of surgical bioprosthesis plays an important role for the procedure planing. Indeed malposition and inappropriate sizing of the sutureless valve are associated to the development of paravalvular leak and early degeneration of the prosthesis (49). In case of malposition the valve is generally well anchored, but the inflow ring is located below or above the native aortic valve annulus. The prosthesis size overestimation can generate premature degeneration of the leaflets and metal structure recoil, reducing contact between prosthesis and aortic wall, and consequent paravalvular leak. Even in the absence of recoil, a slight in-folding of the prosthesis can induce premature degeneration and malfunction of the leaflets, with consequent risk of stenosis and or intraprosthetic regurgitation. The relatively recent experience in the implantation of sutureless bioprosthesis and the consequent low incidence of their degeneration may explain the few available data on sutureless VIV-TAVI procedures. A small prospective registry have demonstrated the feasibility of sutureless VIV-TAVI procedures and the short-term outcomes have noted that mean post procedural transaortic gradient decreased with time after the procedure, suggesting that the dimensions and effective orifice area of the THV kept on growing during the first weeks after implantation (48).

## Conclusions

In the last decades transcatheter techniques have offered different opportunity to treat valve disease and VIV-TAVI represents an emerging treatment of patients with failed surgical biological prosthesis. Indeed 15 years after the first reported VIV case, the VIV-TAVI procedure is now routinely performed worldwide in the vast majority of patients with failed stenotic or regurgitant bioprosthetic valves. Device malposition, ostial coronary obstruction, and high postprocedural gradients represent the most important safety and efficacy concerns of this procedure. Therefore, clinical condition, surgical valve type and anatomy features should be considered to evaluate the better treatment between reoperative SAVR vs. VIV-TAVI to treat failed bioprosthesis. As with any novel emerging therapy, there is a learning curve to the VIV-TAVI procedure and the operator must be aware of the potential challenges. Careful peri-procedural multimodality imaging assessment and procedure planning can help overcome challenges and avoid complications.

## Author Contributions

CA, FBu, and CT conceived the study and performed the procedures, and they critically reviewed the manuscript, giving important intellectual advices. PF made the drawings. SC, PF, ER, FBi, MN, PB, DD'A, AL, and RV collected and interpreted the articles and critically reviewed the manuscript, giving important intellectual advices. All authors contributed to the article and approved the submitted version.

## Conflict of Interest

FBu discloses to have been involved in advisory board meetings or having received speaker's fees from Abbott, Abiomed, Medtronic and Biotronic. CT discloses to have been involved in advisory board meetings or having received speaker's fees from Abbott, Abiomed, Medtronic and Biotronic. CA has been involved in advisory board activities by Abbott, Abiomed, Medtronic and Biotronic. AL discloses to receive speaking honoraria from St. Jude Medical/Abbott, Medtronic, Abiomed and from Bracco Imaging. The remaining authors declare that the research was conducted in the absence of any commercial or financial relationships that could be construed as a potential conflict of interest.

## Publisher's Note

All claims expressed in this article are solely those of the authors and do not necessarily represent those of their affiliated organizations, or those of the publisher, the editors and the reviewers. Any product that may be evaluated in this article, or claim that may be made by its manufacturer, is not guaranteed or endorsed by the publisher.

## Supplementary Material

Supplementary Figure 1 High transcatheter valve implantation inside small failed bioprosthetic valves. Elevated post-procedural gradients has been reported in 26.8% of VIV procedures and they are more common in small surgical valves (≤ 21 mm). High transcatether valve implantation inside failed bioprosthetic valves is a strong independent predictor of lower postprocedural gradients in both self- and balloon-expandable transcatheter valves. A sopranular selfexpandible valve (Evolut R 23) was implanted in a small stented degenerated surgical valve (Mitroflow 19) in a high position [height <5 mm, **(A)**] to avoid post procedural gradient **(B)**.

Supplementary Figure 2 The virtual transcatheter valve to coronary ostium distance (VTC). For assessment of the VTC distance, a virtual ring with the diameter of the fully expanded THV is superimposed onto the short-axis image **(A)**. Compared with the sinus diameter and the coronary ostia height, the VTC distance also accounts for the relative orientation of the bioprosthesis within the aortic root **(B,C)**.

Supplementary Figure 3 Different type of coronary protection. The coronary obstruction is more frequent during VIV procedures (four folders greater compared to TAVI for native aortic valves). The risk of coronary obstruction is also correlated to the type of surgical valve. Indeed it is highest during VIV procedures for surgical bioprothesis such as “stented” bioprostheses that have externally mounted leaflets, and “stent-less” surgical bioprostheses. A more controlled measure, in cases at high-risk for coronary obstruction, is to place a wire in the coronary artery before transcatheter valve implantation, using a guide catheter to approach the coronary ostia from above with not interference with transcatether device implantation [ie, Judkins left, **(A)**]. At the end of transcatether valve implantation the coronary artery flow is good both in selective angiography and in aortography view **(B,C)**. There is no evidence of coronary occlusion risk, the coronary protection is removed without other protection measures. When after the transcatether valve implantation the coronary artery is occluded by surgical valve leaflets **(E)** a stent, previous positioned in the coronary vessel, is implanted at coronary ostium according to chimney/snorkel technique **(D)**. Other option for coronary ostium stenting in case of normal coronary flow but high risk of occlusion by surgical valve leaflets **(E)** is the recannulation and rewiring of the coronary after transcatheter valve release and coronary ostium stenting through the prosthesis valve frame structure, the orthotopic snorkel stenting technique **(F,G)**.

Supplementary Figure 4 The cerebral protection during a valve in valve procedure to reduce the risk of cerebral embolization in more degenerated valve leaflets. A cerebral protection system (Claret Sentinel cerebral protection) was positioned before the procedure by right radial artery **(A)**. After the remove of device **(B)** in the proximal filter debris were found **(C)**.
