# Supplementary figures and images for: Transcatether Aortic Valve Implantation to Treat Degenerated Surgical Bioprosthesis: Focus on the Specific Procedural Challenges
Source: Front Cardiovasc Med. 2022 May 31;9:895477. doi: 10.3389/fcvm.2022.895477 (PMC9194080; doi:10.3389/fcvm.2022.895477)

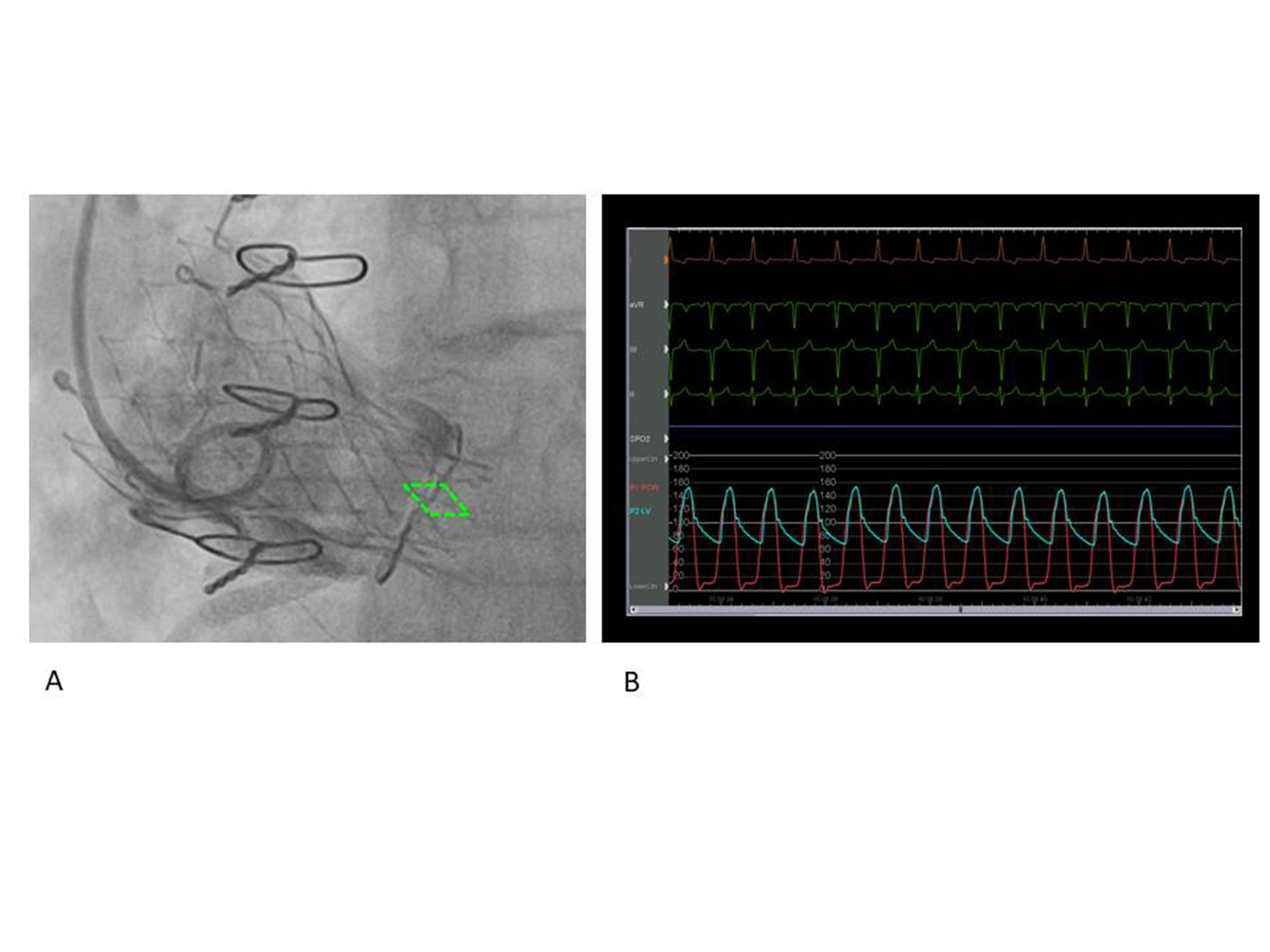

Supplement: Supplementary Figure 1 — High transcatheter valve implantation inside small failed bioprosthetic valves. Elevated post-procedural gradients has been reported in 26.8% of VIV procedures and they are more common in small surgical valves (≤ 21 mm). High transcatether valve implantation inside failed bioprosthetic valves is a strong independent predictor of lower postprocedural gradients in both self- and balloon-expandable transcatheter valves. A sopranular selfexpandible valve (Evolut R 23) was implanted in a small stented degenerated surgical valve (Mitroflow 19) in a high position [height <5 mm, (A)] to avoid post procedural gradient (B). [file Image_1.JPEG]

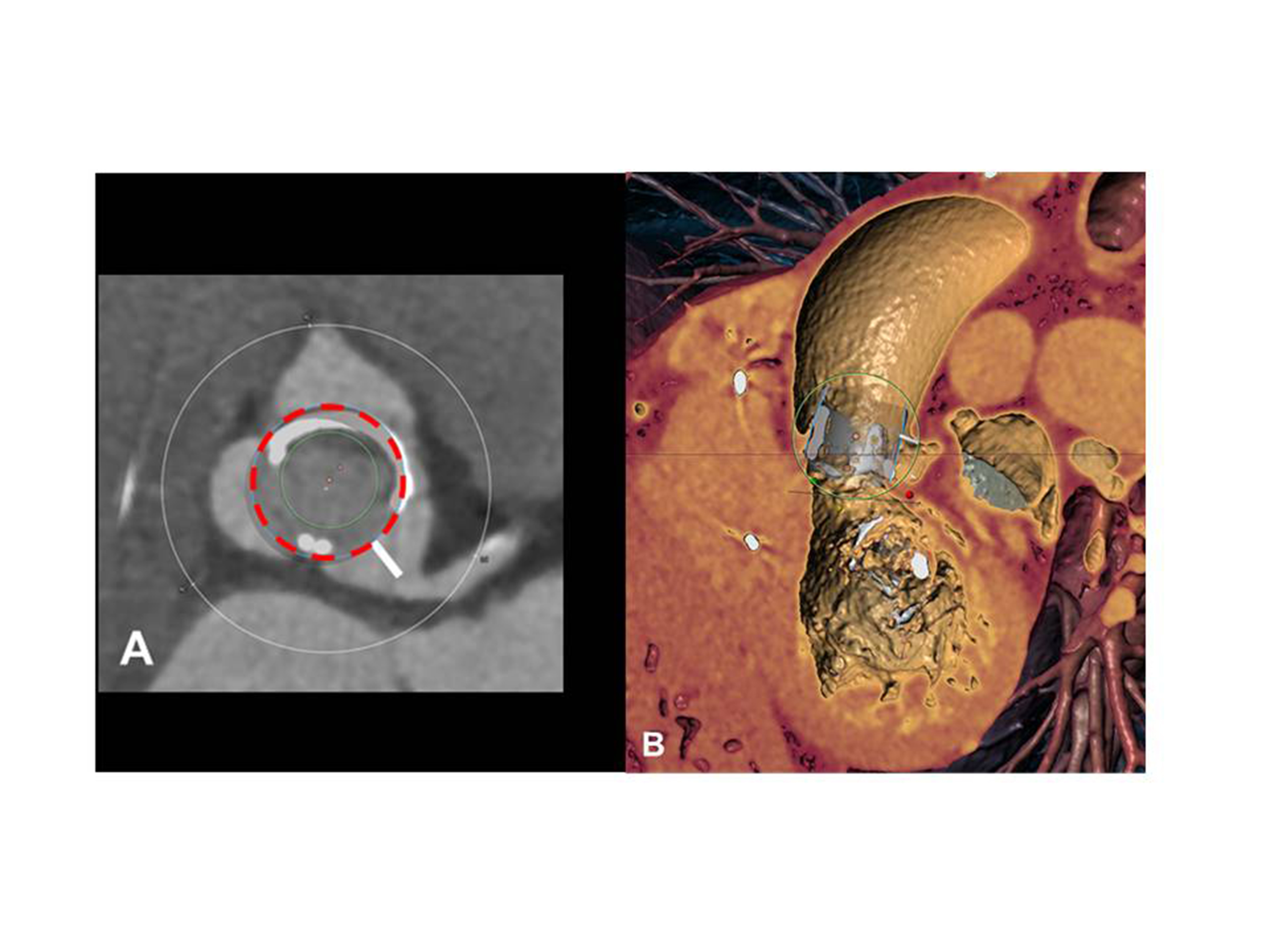

Supplement: Supplementary Figure 2 — The virtual transcatheter valve to coronary ostium distance (VTC). For assessment of the VTC distance, a virtual ring with the diameter of the fully expanded THV is superimposed onto the short-axis image (A). Compared with the sinus diameter and the coronary ostia height, the VTC distance also accounts for the relative orientation of the bioprosthesis within the aortic root (B,C). [file Image_2.JPEG]

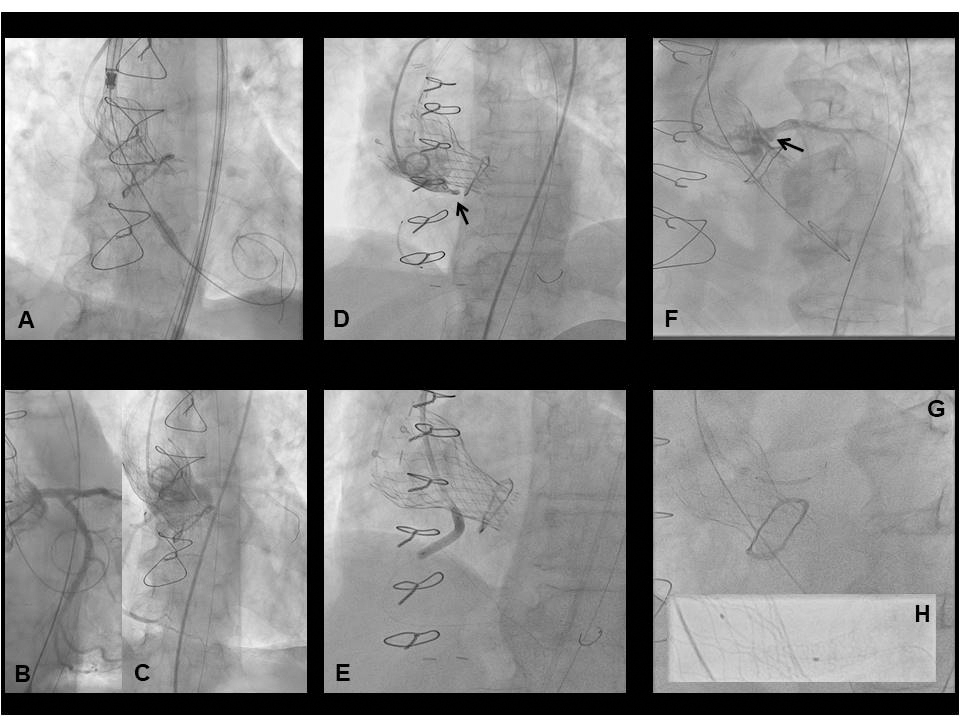

Supplement: Supplementary Figure 3 — Different type of coronary protection. The coronary obstruction is more frequent during VIV procedures (four folders greater compared to TAVI for native aortic valves). The risk of coronary obstruction is also correlated to the type of surgical valve. Indeed it is highest during VIV procedures for surgical bioprothesis such as “stented” bioprostheses that have externally mounted leaflets, and “stent-less” surgical bioprostheses. A more controlled measure, in cases at high-risk for coronary obstruction, is to place a wire in the coronary artery before transcatheter valve implantation, using a guide catheter to approach the coronary ostia from above with not interference with transcatether device implantation [ie, Judkins left, (A)]. At the end of transcatether valve implantation the coronary artery flow is good both in selective angiography and in aortography view (B,C). There is no evidence of coronary occlusion risk, the coronary protection is removed without other protection measures. When after the transcatether valve implantation the coronary artery is occluded by surgical valve leaflets (E) a stent, previous positioned in the coronary vessel, is implanted at coronary ostium according to chimney/snorkel technique (D). Other option for coronary ostium stenting in case of normal coronary flow but high risk of occlusion by surgical valve leaflets (E) is the recannulation and rewiring of the coronary after transcatheter valve release and coronary ostium stenting through the prosthesis valve frame structure, the orthotopic snorkel stenting technique (F,G). [file Image_3.JPEG]

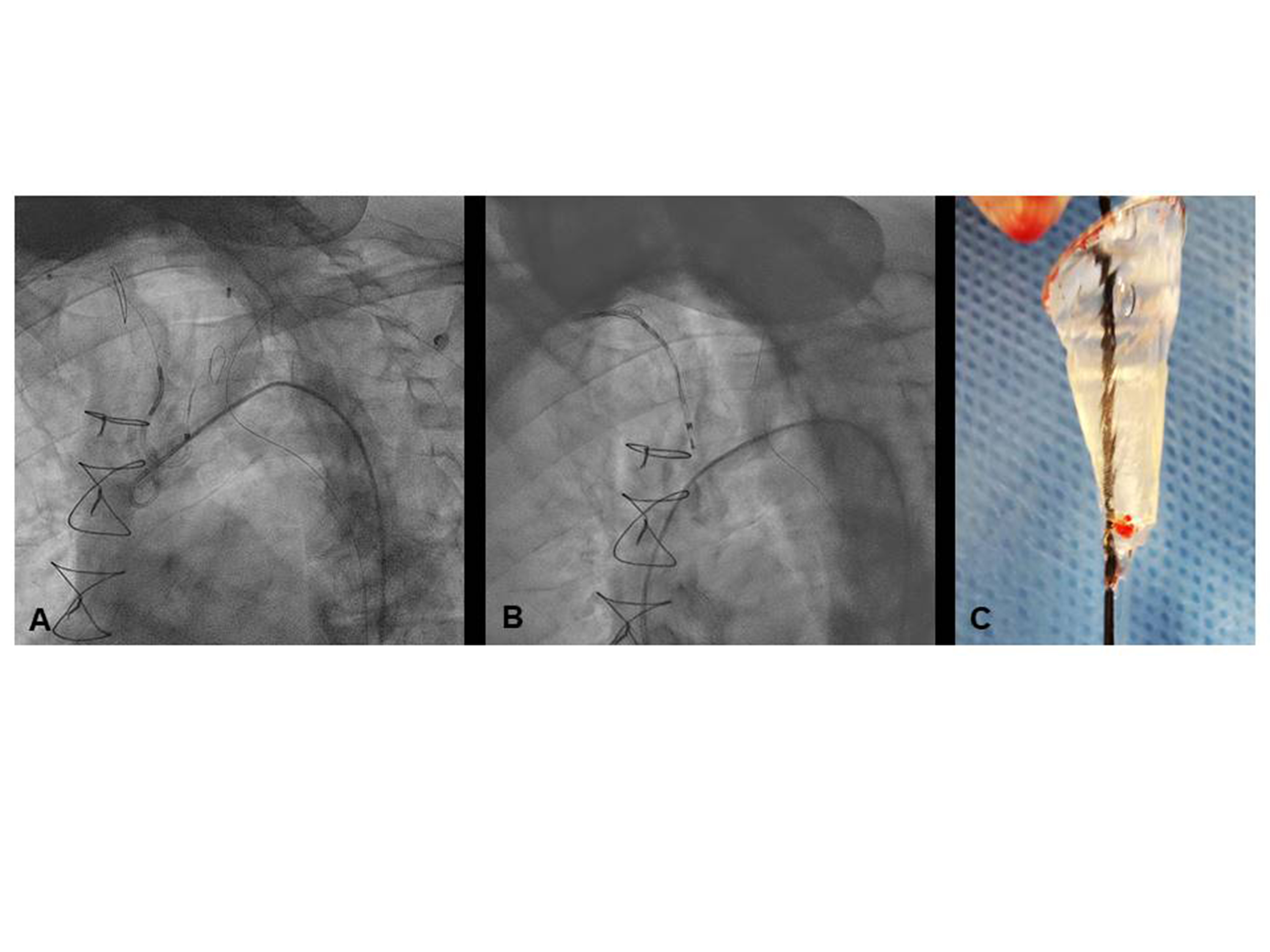

Supplement: Supplementary Figure 4 — The cerebral protection during a valve in valve procedure to reduce the risk of cerebral embolization in more degenerated valve leaflets. A cerebral protection system (Claret Sentinel cerebral protection) was positioned before the procedure by right radial artery (A). After the remove of device (B) in the proximal filter debris were found (C). [file Image_4.JPEG]
